# Biochemical response and nutrient uptake of two arbuscular mycorrhiza-inoculated chamomile varieties under different osmotic stresses

**DOI:** 10.1186/s40529-021-00328-3

**Published:** 2021-12-11

**Authors:** Fatemeh Ebrahimi, Amin Salehi, Mohsen Movahedi Dehnavi, Amin Mirshekari, Mohammad Hamidian, Saeid Hazrati

**Affiliations:** 1 Department of Agronomy and Plant Breeding, Faculty of Agriculture, Yasouj University, Yasouj, Iran; 2 Department of Agronomy, Faculty of Agriculture, Azarbaijan Shahid Madani University, Tabriz, Iran

**Keywords:** *Funneliformis mosseae*, *Matricaria chamomilla*, Nutrient uptake, Water-deficit stress

## Abstract

**Background:**

Water-deficit stress is known as one of the most severe environmental stresses affecting the growth of plants through marked reduction of water uptake, which leads to osmotic stress by lowering water potential. Adopting appropriate varieties using soil microorganisms, such as arbuscular mycorrhiza (AM) fungi, can significantly reduce the adverse effects of water deficiency. This study aimed to evaluate the role of *Funneliformis mosseae* on nutrient uptake and certain physiological traits of two chamomile varieties, namely Bodgold (Bod) and Soroksári (Sor) under osmotic stress. For pot culture, a factorial experiment was performed in a completely randomized design with three factors: osmotic stress (PEG 6000) was applied along with Hoagland solution at three levels (0, -0.4 and -0.8 MPa), two German chamomile varieties (Bodgold (Bod) and Soroksari (Sor)), and AM inoculation (*Funneliformis mosseae* species (fungal and non-fungal)) at four replications in perlite substrate.

**Results:**

Osmotic stress significantly reduced the uptake of macro-nutrients (N and P) and micro-nutrients (Fe, Cu, Mn, and Zn) in the shoots and roots. Moreover, the level of osmolytes (total soluble sugars and proline) and the activity of antioxidant enzymes in the shoots of both varieties increased under osmotic stress. Regarding the Sor variety, the level of these compounds was more satisfactory. AM improved plant nutrition uptake and osmolyte contents while enhancing antioxidant enzymes and reducing the adverse effects of osmotic stress. Under osmotic stress, the growth and total dry weight were improved upon AM inoculation.

**Conclusions:**

In general, inoculation of chamomile with AM balanced the uptake of nutrients and increased the level of osmolytes and antioxidant enzymes; hence, it improved plant characteristics under osmotic stress in both varieties. However, it was found to be more effective in reducing stress damages in the Sor variety.

## Introduction

Water-deficit stress (due to global warming and climate change) is the leading cause of decremented annual plant performance. In arid and semi-arid regions, plants are exposed to water-deficit stress due to the simultaneous increase in the rate of transpiration and temperature, in addition to the reduction in root access to water (Halo et al. 2020). Water-deficit stress affects plant life in many ways; for example, shortage of water to roots reduces the rate of transpiration and induces oxidative stress (Hasanuzzaman et al. 2013). Water-deficit stress imparts deleterious effects on plant growth by affecting enzyme activity, nutrient uptake, and nutrient assimilation (Ahanger et al. 2017).

The use of herbal medicinal products and supplements has tremendously increased over the past three decades. Over 80% of the world’s population relies on these products as a part of their primary healthcare (Sharma, 2004; Ekor 2014). German chamomile (*Matricaria chamomilla* L.) belongs to the family of *Asteraceae*, one of the most common medicinal plants (Wichtl 2004). Chamomile is a prominent medicinal plant whose compounds are considered to be safe (Sharifi et al. 2014). German chamomile flower and its extracts have antimicrobial and antioxidant activity, and have been used as a painkiller, anti-anxiety, antispasmodic, anti-inflammatory, and anti-gastrointestinal agent (Rehmat et al. 2020). With the rise in the global demand for medicinal plants, there is an urgent need to increase their cultivation and production. The increasing demand for medicinal plants, especially chamomile, necessitates deeper knowledge to adopt drought-resistant varieties. In addition to water management, the selection of the right genotype can also contribute to preventing water-deficit stress damages and promoting the sustainable use of water resources. Concerning medicinal plants, although water-deficit stress increases the synthesis of secondary metabolites, it can decline the growth of the plant, particularly its vegetative and reproductive organs (biomass) which generally contain medicinal compounds (Selmar et al. 2017). It also reduces the nutrient uptake of chamomile (Salehi et al. 2016), which plays a vital role in its total dry weight and essential oil content (Andrzejewska and Woropaj-Janczak 2014). Under water-deficit stress, lipids, proteins, and nucleic acids are damaged due to the rise in the content of reactive oxygen species (ROS) (Uzilday et al. 2012). Plants exploit effective systems, such as antioxidants enzymes catalase (CAT*),* superoxide dismutase *(*SOD*),* and ascorbate peroxidase (APX*)* along with osmolytes, such as soluble sugars to combat toxic ROSs and reduce their consequent damages (Al-Arjani et al. 2020).

As one of the commercial varieties of chamomile tetraploid, Bodgold (Bod) has shown a favorable performance in terms of total dry weight and essential oil among the other types of diploid and tetraploid chamomile (Banatska (2x), Lutea (4x), Zloty Lan (4x), and Goral (4x)) (Tsivelika et al. 2018). Soroksári (Sor) is another important diploid variety of chamomile with a desirable essential oil content compared to Lutea, Goral (tetraploid), and Bona (diploid) varieties, according to Gosztola et al. (2010). Heretofore, no comparative studies have explored the activity of antioxidant enzymes, absorption of nutrients, and dry weight of these varieties under stress conditions. Nonetheless, a study reported an increase in proline and antioxidant activity of the Bod variety under water-deficit stress (Benabdellah et al. 2011). Arbuscular mycorrhizal (AM) fungi coexist with the roots of most plants and have exhibited a great potential for counteracting environmental stresses since they can increase the availability of plants to a larger volume of the rhizosphere and also improve water and nutrient uptake (Zhang et al. 2018) via morphological changes of root volume and their hyphae (Hameed et al. 2014). Additionally, these fungi enhance the nutrient uptake by increasing the synthesis of the compounds and enzymes involved in the absorption process, such as phosphatase (Hu et al. 2013). However, there is a strong body of evidence on drought stress alleviation by AM in different crops (Begum et al. 2019).

Improving the nutrient and water absorption by AM promotes plant growth and reduces the adverse effects of water-deficit stress caused by PEG (Benabdellah et al. 2011; Wu et al. 2013). The beneficial effects of AM have been reported in several *Asteracea* families under water-deficit stress; for instance, under water-deficit stress condition, AM improves the absorption of macro and micro-nutriments and increases the total dry weight of plants from the *Asteracea* family, including *Echinacea angustifolia* (Attarzadeh et al. 2019), German chamomile (Benabdellah et al. 2011), *Helianthus annuus* (Gholamhoseini et al. 2013), Marigold (Asrar and Elhindi 2011), Safflower (Abbaspour 2010), and Scabious (*Knautia arvensis*) (Doubková et al. 2013). AM also improves the growth and yield in *Echinacea angustifolia* by increasing the defensive level of antioxidants (catalase and peroxidase) and osmolyte (proline) enzymes (Attarzadeh et al. 2019).

Therefore, the present study aimed to evaluate the effect of *Funneliformis mosseae* on reducing the impact of osmotic stress of the two most important German chamomile varieties to determine the more resistant one, according to their physiological traits and nutrient uptake. Deciphering the AM-mediated mechanisms in the plants’ protection responses and metabolic pathways under unfavorable conditions is required to gain insight into their potential. Furthermore, it will shed light on new approaches to exploiting AM as a bioprotective tool against osmotic stresses in sustainability and food security.

## Materials and methods

### Experimental design

A pot experiment was performed in the factorial arrangement within a completely randomized design with four replications in the research greenhouse of the Faculty of Agriculture at Yasouj University. The greenhouse temperature was 25 ± 2 °C. A soilless cultivation system with perlite, as inert substrate, was developed to study the effect of different osmotic stresses on chamomile plants colonized with the AM *Funneliformis mosseae*. The PEG treatment (osmotic stresses) was applied along with Hoagland solution at three osmotic stresses (control, − 0.4, and − 0.8 MPa). Distilled water was used as the control treatment with Hoagland solution. Different levels of osmotic stress were prepared utilizing polyethylene glycol 6000 (PEG) via the formula proposed by Michel and Kaufman (1973) and applied in nutrient solutions. The second studied factor was the use of arbuscular mycorrhiza (AM) fertilizer (*F. mosseae* species (Zist Fanavar Sabz Company, Iran) (fungal and non-fungal)), which was initially inoculated in the culture medium. In mycorrhizal plants, each pot of mycorrhizal treatment received 40 g of AM inoculant (containing spore numbers of 120 g^−1^ substrate) at a depth of 5 cm and incorporated well within the cultivation bed before transplanting. Two German chamomile varieties (Bodgold (Bod) and Soroksari (Sor)) were considered. The Bod variety seeds were purchased from Isfahan Agricultural and Natural Resources Research Center and the Sor variety was supplied by the Yasouj Zardband Company. The seeds of both varieties were sterilized with sodium hypochlorite solution (1%) for 3 min and then washed several times in distilled water. To produce seedlings, the seeds were first transplanted in a bed of peat moss and cocopeat (1: 2) in a 72-cell (30 cc) seedling tray. At the 4–6-leaf stage, they were transferred to plastic pots with a height of 25 cm and a diameter of 18 cm (6 seedlings per pot), filled with sterilized perlite (sterilized with autoclave (105 °C for 30 min)). After transferring the plants to the pot, the plants were pre-cultured with 1/4 Hoagland’s nutrient solution, which contained phosphorus: 7.75 mg L^−1^ for 4 weeks to adapt- and establish the seedlings to the new condition. During the osmotic stress phase, the modified Hoagland’s nutrient solution was used in this protocol, which contained phosphorus: 31 mg L^−1^, 5 mM KNO_3_, 5 mM Ca(NO_3_)_2_, 1 mM NH_4_H_2_PO_4_, 2 mM MgSO_4_, 0.4 mM H_3_BO_3_, 0.08 mM MnCl_2_, 1.8 μM ZnSO_4_, 0.1 μM (NH4)_6_ Na_2_MoO_4_, 3 μM CuSO_4_, and 0.1 mM FeSO_4_ (Hoagland and Arnon 1950).

The osmotic stress treatments started 4 weeks after transplanting, and osmotic stress was gradually applied and maintained for 4 weeks. The pH of the solutions was adjusted to 5.8 ± 0.1 prior to each irrigation. It should be noted that all the seed-starting containers, pots, and seedling beds were decontaminated with a greenhouse autoclave, and distilled water was used to make all the nutrient solutions.

### Sampling to measure dry weight, physiological, and nutrients traits

28 days following osmotic stress, two plants per pot were harvested and pooled as one replicate (four replicates for each treatment), and young upper leaves were sampled to measure the physiological traits. The samples were transferred to the laboratory after being placed in a liquid nitrogen container, where they were stored at − 40 °C. To measure the traits associated with the dried sample, four plants were harvested from each pot, pooled as one replicate, and dried at 70 °C for 48 h.

### Mycorrhizal determinations

The plants were harvested 56 days following inoculation, and the percentage of mycorrhizal root colonization was estimated by visual observation of fungal colonization after clearing the washed roots with distilled water and cutting them into segments with a length of 1 cm. Approximately 100 root segments were randomly chosen and cleared in 10% KOH, after which they were placed in a water bath at 90 °C for 30 min and stained with 0.05% Trypan blue in acetic acid (v/v), according to Cao et al. (2013). The rate of mycorrhizal colonization was estimated by Biermann’s and Linderman (1981) method.

### Enzyme activity

To prepare the enzymatic extract, 3 ml of extraction buffer (100 mM potassium phosphate at pH = 7.8, 0.1 M EDTA, and 0.1 M PVP) was homogenized with 0.1 g of leaf sample using a mortar in an ice bath. The obtained homogeneous samples were centrifuged for 30 min (14,000 rpm at 4 °C) and the supernatant was used to measure the enzymes activity. Catalase (CAT) activity was evaluated by monitoring the reduction in the absorption of hydrogen peroxide in the reaction mixture at 240 nm employing a spectrophotometer (Aebi 1984). Peroxidase (POD) activity was also assessed according to the absorption of the reaction mixture (enzymatic extract, potassium phosphate buffer, guaiacol, along with 30% H_2_O_2_) at 470 nm (Zhou and Leu 1999). Polyphenol oxidase (PPO) activity was measured based on the intensity of the orange color of methyl catechol at a wavelength of 420 nm produced in the reaction mixture (Kahn 1975). The CAT, POD, and PPO activity of the extract was expressed as enzyme unit mg^−1^ protein min^−1^**.** One unit of enzyme activity is defined as the amount required to decompose µl mol of the substrate within one min.

### Determination of proline content

To determine the amount of proline in the shoot, 0.1 g of fresh tissues were homogenized with 10 ml of 3% aqueous sulfosalicylic acid followed by centrifugation. We blended 2 ml of the supernatant with acid ninhydrin and glacial acetic acid (2 ml of each). The mixture was kept in a water bath for 1 h at 100 °C. Subsequently, the reaction mixture was extracted with toluene (4 ml), whose absorbance was determined at 520 nm after cooling down to the room temperature (Paquin and Lechasseur 1979).

### Determination of the total soluble sugar

The total soluble sugar was determined via the method specified by Irigoyen et al. (1992). Fresh leaves (0.1 g) were added to 5 ml of 80% ethanol in a water bath and heated for 1 h at 80 °C. Afterwards, 1 ml of the sample extract was taken to another set of test tubes and mixed with 1 ml each of 18% phenol and distilled water. They were then remained at the room temperature for an hour. Finally, 5 ml of sulfuric acid was added and the whole mixture was vortexed. The absorbance was read at 490 nm with a UV spectrophotometer. Ethanol 80% was used as a blank sample. Absorbance was recorded at 625 nm utilizing a spectrophotometer.

### Measurement of nutrients

The extract for measuring the nutrients was prepared based on digestion via the H_2_SO_4_-salicylic acid-H_2_O method. This extract was employed to measure the concentrations of nitrogen (N) (Novozamsky et al. 1974), manganese (Mn) (Atomic Absorption), potassium (K) (Film Photometer) (Knudsen et al. 1982), and phosphorus (P) (Røtset 1985). To measure iron (Fe), zinc (Zn), and copper (Cu), the samples were placed at 500 °C for 4 h, and 5 ml of two normal hydrochloric acids (2n) was added to them. The samples were then placed on a heater. The concentration of Fe, Zn, and Cu in the shoot and the roots of the plant was determined through the atomic absorption (Chapman and Pratt 1961).

### Statistical analysis

All the analytic determinations were carried out in quadruplicate. The main effects of the interaction among the experimental factors were determined via the analysis of variance (ANOVA) using a general linear model. Subsequently, the means were separated with a least significant difference (LSD) test with *P* < *0.05* utilizing the SAS software 9.1 (SAS Institute, Cary, NC, United States); the graphs were drawn with Excel 2013 software. The comparison of the means was performed using the LSD at a *P*-value of 5%. In case of a significant interaction, the LS means procedure was used to compare the interactions. When an *F*-test indicated statistical significance at *P* < 0.05, the protected least significant difference was used to separate the means of the main effect, and the significant interactions were separated applying the slicing method. Once the interactions were not significant, we only discussed the main effects. In case the main effects and the two-way and the three-way interaction effects of the traits were significant, we only discussed the three-way interaction effects or when the main effects, or two-way interaction traits were significant, we only discussed the two-way interaction effects. Pearson correlation analysis was carried out by the XLSTAT (Addinsoft, Paris, France) program.

## Results

### Root colonization

**Fig. 1:**
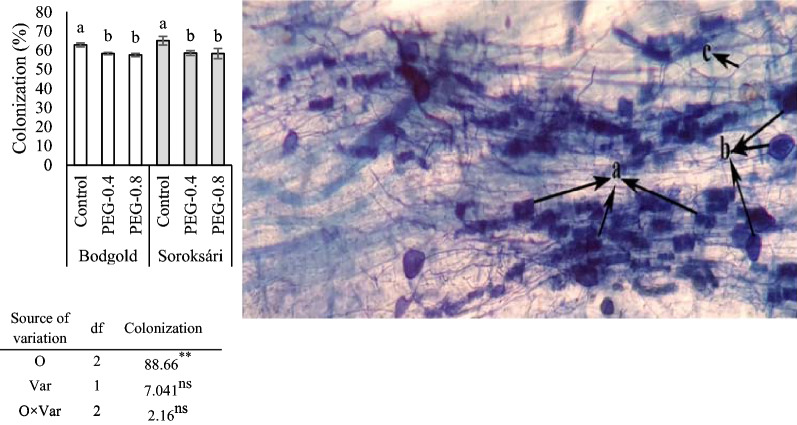
Structures of Arbuscular Mycorrhizal Fungi (AM) infection rate in chamomile root, the stainedin figure includes the components of mycorrhizae *a* Arbuscules, *b* Vesicles, *c* internal hyphae (**A**). Colonization rate of mycorrhizal *F. mosseae* under osmotic stress (**B**)

At the end of the experiment, no AM colonization was found in the roots of the non-inoculated chamomile varieties of the seedlings. Colonization assay results, represented in Fig. 1, showed that the plant roots were significantly colonized by AMF as compared to those of the un-inoculated control treatments under osmotic stress. In the stained root segments, the root mycorrhizas had clearly visible intraradical arbuscules, vesicles, and internal hyphae (Fig. 1). Osmotic stress reduced the mycorrhizal colonization in both varieties. The rate of root colonization was similar between the two varieties. Moreover, colonization in the control treatment was about 9% higher than the -0.4 MPa while its rate did not significantly differ between the − 0.4 and − 0.8 MPa treatments (Fig. 1).

### Concentration of macro-nutrients (N, P, and K) in roots and shoots

**Fig.2:**
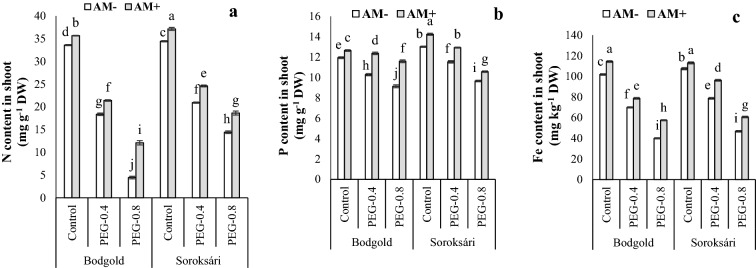
Interaction effect of osmotic stress (control, − 0.4, and − 0.8 MPa) × arbuscular mycorrhiza (AM) × variety on N content in shoot (**a**), P content in shoot **b** and Fe content in shoot **c** of chamomile. Means within a column followed by the different letter are significantly different at *P* ≤ 0.05. Standard error of the mean (n = 4)

The three-way interaction between different levels of osmotic stress, mycorrhiza, and variety was significant on the nitrogen (N) and phosphorus (P) concentrations of the shoots (Table 1). However, the P and N concentrations of the roots and the potassium (K) level of the shoots and roots were evaluated based on their significance at 5% level as interaction and main effects (Table 1). Osmotic stress reduced N accumulation in the shoots. High levels of osmotic stress (− 0.8 MPa) significantly declined the uptake of these nutrients in the Bodgold (Bod) variety. Regardless of AM application, the N concentration of the shoots of the Soroksári (Sor) variety was much higher than that of the Bod variety. Nonetheless, the difference became more evident under osmotic stress; accordingly, the N concentrations of the shoots of Bod were 4% lower than those of the Sor variety for AM + treatment under normal conditions. Under stress at osmotic stresses of − 0.4 and − 0.8 MPa, AM-inoculated roots this value reached 13 and 35%, respectively (Fig. 2). The osmotic stress also reduced the N concentration of the roots, but AM partially increased the concentration of this uptake of the nutrient in the roots under both stress and control conditions. Additionally, the N concentrations of the roots of both varieties were almost the same. However, the effect of AM on the increase in the concentration of this nutrient was higher in Sor as compared with that in the Bod variety (Table 2). AM reduced the adverse effects on the P concentration of the shoots. At the osmotic stress of − 0.4 and − 0.8 MPa, the impact of AM on the amount of P in the shoots was stronger than that in the Bod variety. In the AM + treatment, the P concentration in the shoots of the Bod variety was 20.5 and 27.5% higher at stress levels of − 0.4 and − 0.8 MPa, respectively. In case of the Sor variety, this parameter was 12 and 10% higher compared to the AM- treatment. In general, the P level of the shoot of the Bod in the Sor variety was lower than that in the Sor variety; thus, the highest P concentration of the Sor variety shoots was observed upon AM inoculation (Fig. 2). The P concentration of the roots also decreased with the reduced amount of osmotic stress. The P amount of the roots was higher in the Sor variety. With the increase in stress, the mentioned difference declined as the P concentration of the Sor roots was 25% under the control condition, which decremented to 11 and 3% at osmotic stresses of − 0.4 and − 0.8 MPa, respectively (Table 2). According to Table 2, AM caused a 12% enhancement in the P concentration of the roots. Osmotic stress increased the K level of the shoots and roots. Under non-stress conditions, the K concentration of the shoots of the Sor variety was 10% higher than that of the Bod variety. With the rise in the osmotic stress, the difference narrowed and the K concentration of the shoots were not therefore significantly different between the two varieties at the osmotic stress of -0.8 MPa. According to the mean comparison of the main effects of the treatments (Table 2), AM generally increased the K concentration of the shoots in the chamomile by 9%. Both osmotic stress and AM treatments enhanced the K concentration of the roots whereas the Sor variety exhibited higher root K concentration. As already stated, AM increased the root K concentration. Under stress conditions, this increase was more profound; therefore, the potassium concentrations of the AM-inoculated roots were respectively 17, 25, and 19% higher than those in the non- AM- treatment under normal (control) and osmotic stress of − 0.4 and − 0.8 MPa (Table 2).

**Table 1:** Analysis of variance of osmotic stress, arbuscular mycorrhiza and variety and their interactions effects on nutrients uptake in chamomile

Source of variation	df	Shoot concentration							Root concentration						
		N	P	K	Zn	Fe	Cu	Mn	N	P	K	Zn	Fe	Cu	Mn
O	2	2114.47**	29.81**	1565.17**	65040.23**	13444.14**	2636.89**	3939.09**	164.26**	131.61**	2966.65**	51988.02**	428.06**	4332.16**	2803.51**
AM	1	181.96**	25.66**	208.29**	4504.68**	1906.38**	2813.66**	3236.61**	41.25**	26.8**	506.32**	3663.02^ns^	453.25**	1328.25**	1652.08**
Var	1	201.80**	5.32**	55.60**	500.52**	550.13**	338.66**	413.24*	1.28**	26.1**	606.16**	1518.65**	56.42**	10.50**	42.18**
O×AM	2	13.38**	0.65**	0.041^ns^	289.45**	43.88**	9.66**	26.66^ns^	3.32**	0.66^ns^	31.50**	25.52^ns^	32.16**	4.03^ns^	115.65**
O×Var	2	54.56**	2.6**	22.20**	111.84*	129.06**	60.60^ns^	196.69*	0.21^ns^	3.69**	18.66**	465.00**	1.14**	22.68*	22.26**
AM×Var	1	1.58**	0.6**	2.98^ns^	133.33^ns^	1.16	141.69*	269.56*	0.62	0.30^ns^	19.65**	4.68^ns^	0.13^ns^	1.16^ns^	33.33**
O×AM×Var	2	5.46**	1.06**	5.08^ns^	28.28^ns^	68.35**	50.39^ns^	80.29^ns^	0.16^ns^	0.35^ns^	0.69^ns^	4.68^ns^	0.91^ns^	5.06^ns^	6.38^ns^
Error	36	0.223	0.019	2.46	34.11	1.16	28.68	45.64	0.146	0.30	2.62	14.40	1.51	4.46	4.06
CV%		2.05	1.20	3.15	3.69	1.34	8.34	10.23	4.63	5.08	4.64	3.12	3.26	3.98	3.66

*O* Osmotic stress, *Var* Varieties, *AM* Arbuscular mycorrhizal inoculation, *ns* non-significance at *P* ≤ 0.05; ^*^P ≤ 0.05; ^**^P ≤ 0.01, statistical significance

**Table 2:** Interaction effect of osmotic stress with variety, osmotic stress with arbuscular mycorrhiza, arbuscular mycorrhiza with variety and main effect of arbuscular mycorrhiza on macro nutrients uptake of shoot and root of chamomile

		Treatments		Shoot concentration	Root concentration		
				K (mg g^−1^ DW)	N (mg g^−1^ DW)	P (mg g^−1^ DW)	K (mg g^−1^ DW)
Two-way interactions	O × Var	Control	Bod	36.5e	10.7b	9.7e	18.6f
		PEG-0.4	Bod	51.8c	8.6c	13.2c	30.1d
		PEG-0.8	Bod	58a	4.5e	16.5a	43.7b
		Control	Sor	41d	11.3a	12.2d	24.33e
		PEG-0.4	Sor	54b	8.7c	14.7b	38.4c
		PEG-0.8	Sor	57.8a	4.9d	17a	53.7a
	O × AM	Control	Am +	40.8d	11.9a	11.5e	23.1e
		PEG-0.4	Am +	55.0b	10.1b	14.9c	38.1c
		PEG-0.8	Am +	60.0a	5.2d	17.6a	53.0a
		Control	Am−	36.7e	10.2b	10.5f	19.8f
		PEG-0.4	Am−	50.8c	7.2c	13.1d	30.5d
		PEG-0.8	Am−	55.7b	4.1e	15.8b	44.4b
	AM × Var	Am +	Bod	51.1b	8.8b	13.8b	33.4c
		Am-	Bod	46.4d	7.1c	12.5c	28.2d
		Am +	Sor	52.7a	9.3a	15.5a	42.7a
		Am−	Sor	49.1c	7.2c	13.8b	34.9b
Main effects	AM	Am +		51.9a	9.0a	14.7a	38.1a
		Am−		47.8b	7.2b	13.1b	31.6b

Water-deficit stress at three osmotic stresse*s* (control, − 0.4, and − 0.8 MPa). Plants inoculated (AM +) or not inoculated (AM−) with the arbuscular mycorrhiza fungus *F. mosseae*, two German chamomile varieties (Bodgold (Bod) and Soroksari (Sor)). Means followed by common letter are not significantly different at the level of 5% (LSD test)

### Concentration of micronutrients (Fe, Zn, Mn, and Cu) in roots and shoot

The three-way interaction between different osmotic stress levels, AM, and varieties was significant only on the iron (Fe) concentration of the shoots (Table 1). However, the comparisons between Fe, zinc (Zn), copper (Cu), and manganese (Mn) concentrations of the roots and other micro-elements in the shoots were evaluated based on the significance level of 5% for the main effects and the two-way interaction effects (Table 3). Osmotic stress reduced the Fe concentration of the shoots in both varieties; however, AM significantly increased the amount of this nutrient at all the levels. In comparison with AM-, AM + enhanced the Fe concentration of the shoots of the Bod variety by 12, 12.5, and 44% under osmotic stresses of 0, − 0.4, and − 0.8 MPa, respectively. Moreover, the Fe concentration of Sor correspondingly increased by 5, 22, and 29% (Fig. 2). AM also incremented the accumulation of Fe in the roots of these plants under stress conditions. On the other hand, the Fe concentrations of the roots of the two studied varieties were not significantly different under normal conditions. The reduction in the osmotic stresses declined the Fe concentration of the roots in both varieties; nonetheless, its effect was more significant on the Bod variety (Table 3).

**Table 3:** Interaction effect of osmotic stress with variety*,* osmotic stress with arbuscular mycorrhiza, arbuscular mycorrhiza with variety, and main effect of osmotic stress, arbuscular mycorrhiza on micro nutrients uptake of shoot and root of chamomile

				Shoot concentration			Root concentration			
		Traetments		Zn (mg.kg^−1^ DW)	Mn (mg.kg^−1^ DW)	Cu (mg.kg^−1^ DW)	Fe (mg.kg^−1^ DW)	Zn (mg.kg^−1^ DW)	Mn (mg.kg^−1^ DW)	Cu (mg.kg^−1^ DW)
Two-way interactions	O × Var	Control	Bod	94.7e	74.6a	74.7	53.4a	182.2b	64.1b	64.6b
		PEG-0.4	Bod	227.5b	66.9b	60.3	36.6c	73.4e	53.4d	55.9d
		PEG-0.8	Bod	142.5c	47.8c	49.7	19.7e	92.5d	40.0e	34.0e
		Control	Sor	102.5d	78.1a	78.8	53.4a	188.4a	68.1a	67.1a
		PEG-0.4	Sor	238.4a	80.6a	70.3	40.9b	77.2e	55.6c	61.5c
		PEG-0.8	Sor	143.1c	48.1c	51.6	21.9d	116.3c	39.4e	35.0e
	O × AM	Control	Am +	110.0e	83.6a	84.4a	55.9a	192.8a	74.1a	71.6a
		PEG-0.4	Am +	237.8a	83.4a	73.8b	43.4c	84.4e	57.5b	63.4b
		PEG-0.8	Am +	155.6c	55.6c	57.5c	22.8e	114.4c	46.9d	40.0e
		Control	Am-	87.2f	69.1b	69.1b	50.9b	177.8b	58.1b	60.3c
		PEG-0.4	Am-	228.1b	64.1b	56.9c	34.1d	66.3f	51.6c	54.1d
		PEG-0.8	Am-	130.0d	40.3d	43.8d	18.8f	94.4d	32.5e	29.1f
	AM × Var	Am +	Bod	162.9b	68.9b	67.5b	39.6b	125.2b	57.7b	56.7b
		Am−	Bod	146.9c	57.3c	55.6c	33.5d	106.9d	47.3c	46.5d
		Am +	Sor	172.7a	79.6a	76.3a	41.9a	135.8a	61.3a	60.0a
		Am−	Sor	150.0c	58.3c	57.5c	35.6c	118.8c	47.5c	49.2c
Main effects	O	Control		98.6c	76.4a	76.7a	53.4a	185.3a	66.1a	65.9a
		PEG-0.4		233.0a	73.8a	65.3b	38.8b	75.3c	54.5b	58.8b
		PEG-0.8		142.8b	48.0b	50.6c	20.8c	104.4b	39.7c	34.5c
	AM	AM + AM−		167.8a	74.2a	71.9a	40.7a	130.5a	59.5a	58.3a
				148.4b	57.8b	56.6b	34.6b	112.8b	47.4b	47.8b

Osmotic stress reduced the Cu concentration of the shoots (Table 3). Under normal conditions, there were no significant differences between the Mn concentrations of the shoots of the two varieties. The shoots of the Bod variety showed a decline in Mn at − 0.4 and − 0.8 MPa compared to those in the control. The reduction in this nutrient was observed in the shoot of the Sor variety only at the le− 0.8 MPa. The Mn and Cu levels of the shoots in both varieties did not significantly differ, but AM increased the concentration of both nutrients with a higher rate of increase in the shoot of the Sor variety. Osmotic stress reduced Mn and Cu concentrations of the root. Under both stress and normal conditions, the level of these nutrients was higher in Sor root (Table 3). AM also increased the Zn level of the shoot under osmotic stress conditions. The Zn concentration of the shoots was higher at osmotic stresses of − 0.4 and − 0.8 MPa as compared with that in the control condition. Regarding the root, a reverse trend was observed, and under osmotic stress, the root concentration of these nutrients was less than that in the controls. In addition, the Zn level of the shoot was higher in the Sor variety under all conditions; however, this was significant in normal and osmotic stress of − 0.4 MPa (Table 3). AM inoculation increased the Zn concentration of chamomile by 16% (Table 3). There were no significant differences between the Mn concentrations of the shoots of the two varieties under normal conditions. A decrease was, however, observed in the Mn level of the Bod at all the stress levels. Nonetheless, the decrease in Mn concentration of the shoot of Sor was observed only at the stress level of -0.8 MPa. AM enhanced the Mn and Cu levels of the shoots of the Sor and Bod varieties although their difference was not significant. Osmotic stress reduced Mn and Cu concentrations of the root, but the level of these nutrients was higher in Sor roots than that in Bod under the stress and control conditions. AM also increased the Zn concentration of the shoot under osmotic stress conditions. The results revealed that the Zn amount of the shoot was higher than that of the controls at osmotic stresses of − 0.4 and − 0.8 MPa. Concerning the root, the trend was the opposite as the amount of these nutrients in the root was lower than that in the controls. Furthermore, the Zn concentration of Sor shoot was higher than that of Bod under all the conditions, which was significant under normal and osmotic stresses of -0.4 MPa.

### Osmolytes

**Fig.3:**
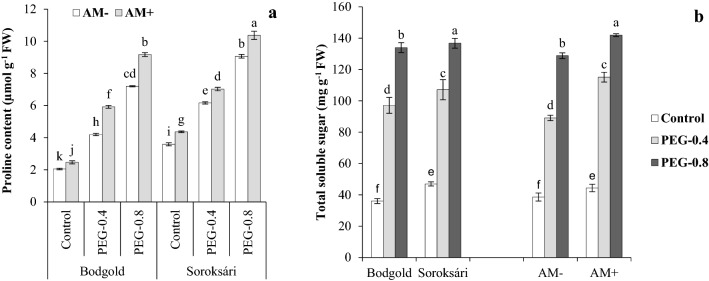
Interaction effect of osmotic stress (control, − 0.4, and − 0.8 MPa) × arbuscular mycorrhiza (AM) × variety and on proline **a** and total soluble sugar **b** content of chamomile. Means within a column followed by the different letter are significantly different at *P* ≤ 0.05. Standard error of the mean of total soluble sugar (n = 6) and proline (n = 4)

According to the results (Table 4), the three-way interaction of variety, osmotic stress and AM significantly affected the proline level whereas the amount of the total soluble sugar was affected by the interaction of osmotic stress × variety and osmotic stress × AM. The osmotic stress generated by PEG increased the levels of proline and total soluble sugar in both varieties. The highest level of these osmolytes belonged to the osmotic stress of − 0.8 MPa. The proline level of Sor was higher than that of Bod variety in all the treatments. Additionally, under stress and non-stress conditions, the total soluble sugar content of Sor was more than that of Bod. Under stress conditions, AM increased the amount of proline in both varieties; however, its effect on the increase in the osmolyte was more evident in the Bod variety. An increase was also observed in the amount of the total soluble sugar of the AM-inoculated samples at different osmotic stresses (Fig. 3).

**Table 4:** Analysis of variance of osmotic stress, arbuscular mycorrhiza and variety and their interactions effects on osmolytes, activity of antioxidant enzymes (catalase (CAT), peroxidase (POD), polyphenol oxidase (PPO)), and shoot and root dry weights

Source of variation	df	Proline	Total soluble sugar	CAT	POD	PPO	Shoot dry weight	Root dry weight
O	2	136.50**	36248.390**	3864.92**	2.62**	217.80**	1.09**	0.002**
AM	1	16.54**	2693.25**	281.51**	0.13**	36.63**	0.083**	0.008**
Var	1	30.56**	753.58**	210.45**	0.34**	33.87**	0.015**	0.00086**
O × AM	2	1.12**	419.88**	26.07**	0.009**	4.40**	0.017**	0.00083**
O × Var	2	0.04**	79.14**	43.14**	0.00**	6.19**	0.148**	0.0001**
AM × Var	1	0.44**	6.87^ns^	2.59^ns^	0.01**	1.35**	0.0068**	0.0003**
O × AM × Var	2	0.43**	17.17^ns^	8.18**	0.013**	0.9**	0.0058**	0.00009^ns^
Error	36	0.006	7.40	0.832	0.0003	0.126	0.0001	0.00003
CV%		1.32	2.92	4.41	3.46	5.08	2.25	5.88

*O* Osmotic stress, *Var* Varieties, *AM* Arbuscular mycorrhizal inoculation, *ns* non-significance at *P* ≤ 0.05; *P ≤ 0.05; **P ≤ 0.01, statistical significance

### The activity of antioxidant enzymes

**Fig.4:**
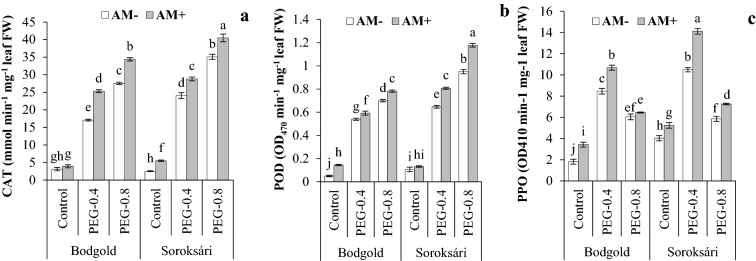
Interaction effect of osmotic stress (control, − 0.4, and − 0.8 MPa) × arbuscular mycorrhiza (AM) × variety and on catalase (CAT) (**a**), peroxidase (POD) **b** and polyphenol oxidase (PPO) (c) activities of chamomile. Means within a column followed by the different letter are significantly different at *P* ≤ 0.05. Standard error of the mean (n = 4)

The three-way interaction of the osmotic stress, AM, and variety was significant on the levels of POD, PPO, and CAT enzymes (Table 4). Osmotic stress increased the activity of all the three enzymes compared to the non-stress conditions. Under stress conditions, the activities of CAT and PPO were higher in Sor as compared with those in Bod variety. Under stress conditions, the activity of the POD enzyme in the Sor variety was higher. AM also enhanced CAT and POD under the control conditions as well as osmotic stress levels of − 0.4 and − 0.8 MPa. The uppermost activity of these enzymes was observed in both varieties upon AM inoculation under the stress potential of -0.8 MPa. For both varieties, the uppermost activity of these enzymes belonged to AM inoculation and the stress potential of − 0.8 MPa. Although the level of PPO enzyme in the stress condition was higher than that in the control, the highest activity was observed under osmotic stress conditions (-0.4 MPa) in the AM-inoculated plants (Fig. 4).

### Dry weight of the roots and shoots

**Fig. 5:**
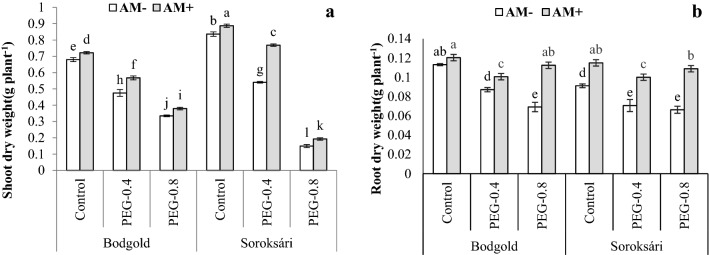
Interaction effect of osmotic stress (control, − 0.4, and − 0.8 MPa) × arbuscular mycorrhiza (AM) × variety and on dry weight of shoot (a), root (**b**). Means within a column followed by the different letter are significantly different at *P* ≤ 0.05 (LSD). Standard error of the mean (n = 4)

A significant three-way interaction was observed regarding osmotic stress, AM, and variety on the dry weight of the roots and the shoots (Table 4). The osmotic stress reduced the dry weight of the root and shoot (Fig. 5). On the other hand, AM + decremented the adverse effect of osmotic stress and increased root dry weight. AM + treatment, at all the levels, led to a higher shoot and root dry weight as compared with AM- treatment. In general, AM improved the dry weight of chamomile at different osmotic stress levels. With the decrease the osmotic stress, shoot dry weight declined; thus, the highest shoot dry weight was obtained in PEG 0, AM + , and the Sor variety (Fig. 5).

**Fig. 6:**
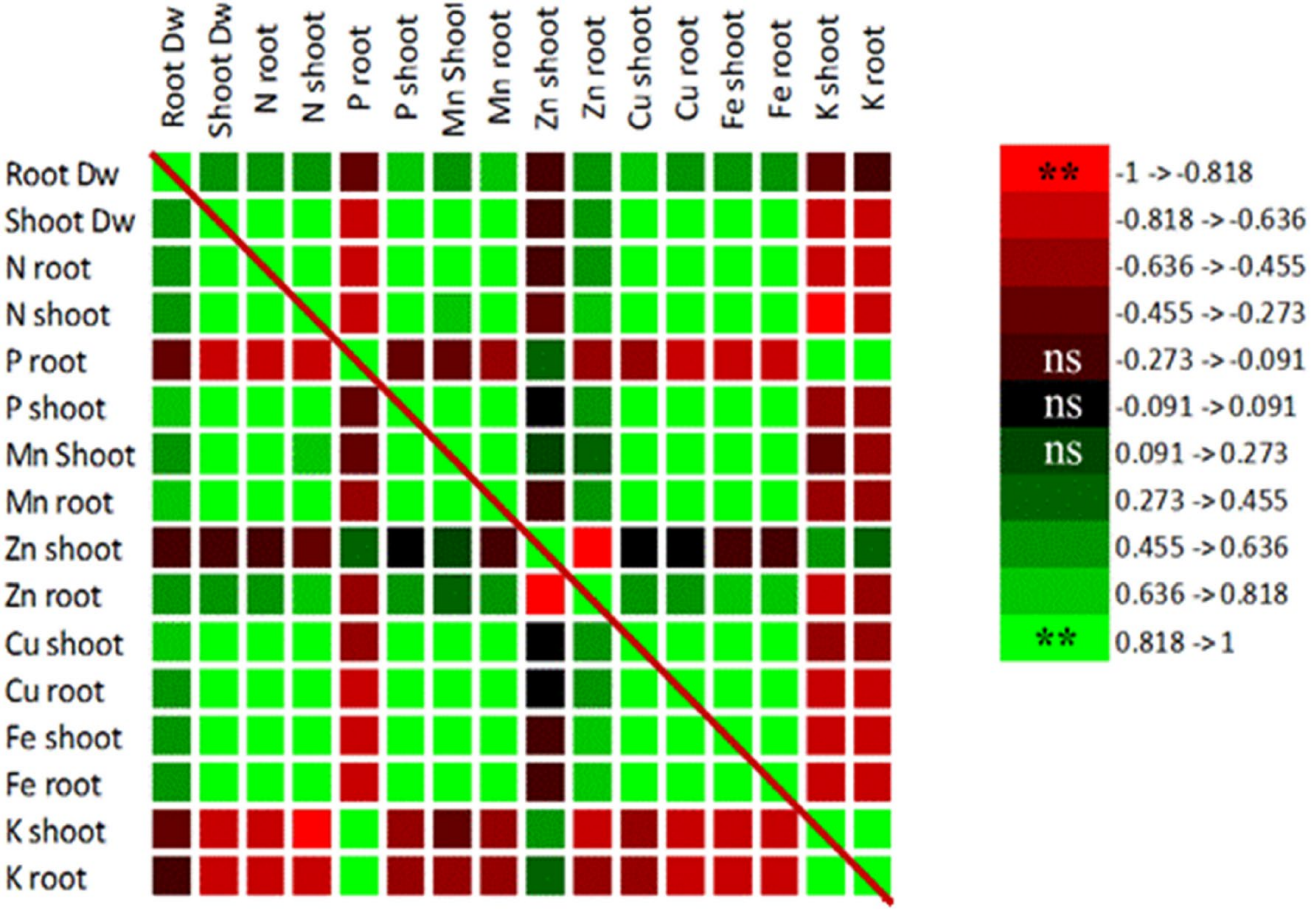
Correlation coefficients among nutrients uptake and root and shoot dry weight (DW). *ns* non-significance; **Significant at 0.01 probability levels

The correlation analysis (Fig. 6) showed a strong correlation between different nutrients uptake and the dry weight of chamomile. The data under normal and osmotic stress conditions with and without AMF colonization were used for correlation analysis; for example, P and N concentrations of the shoot and Mg, Fe, and Cu levels of the roots showed a slightly positive and significant correlation with shoot dry weight. In chamomile, the concentration of each nutrient indicated certain relations with the uptake of other nutrients, the majority of which were synergistic (positive); for example, a slightly positive and significant correlation was observed between the Mg, P, N, Cu, and Fe nutrients of the shoot. Moreover, the Mg and P concentrations of the root, in addition to being highly correlated with each other, showed a positive correlation with other nutrients uptake, such as N, Fe, and Cu of the shoots and roots. Even though the effects of the nutrients on each other were more synergistic, a significant negative correlation was also observed between the shoot and root, and between K and N concentrations of the shoot.

## Discussion

**A** soilless cultivation system with perlite as substrate was developed to study the osmotic stresses in a nutrient solution by non-mycorrhized and AM-colonized chamomile varieties plantlets. The system was adequate for both chamomile varieties plantlet and its fungal associate *F. mosseae*. The results revealed that AM fungi inoculation with chamomile varieties seedlings could improve osmotic stress tolerance of host plants under stress condition, which is a very important piece of information for under-drought areas in the world. These findings indicated that to reduce the unfavorable effects of osmotic stress on chamomile plant growth, the use of AM inoculation ought to be considered as a biological method to alleviate chamomile stress.

The colonization rate of mycorrhizal fungi reflects the degree of infection and affinity between AM and the host plant. In the present study, under all the osmotic stresses, the AM colonization rate was over 57%, indicating a relatively high affinity between the selected AM and chamomile varieties. Under the osmotic stresses, however, mycorrhizal colonization rate decreased significantly, which probably had an impact on the microbial activities, and affected the function of AM symbionts to a certain extent. This decrease in colonization was due to water shortage in the studied pots since environmental factors strongly affect colonization. The decrease may also be due to the low carbon availability in the host plants under drought stress, or because drought stress could have inhibited spore germination and hyphal growth in the rhizosphere soil (Chen et al. 2020). Wu and Xia (2006) found that drought stress significantly decreased the mycorrhiza colonization of *Glomus versiforme*. They suggested that arid and semiarid environments had adverse effects on mycorrhiza fungi development in host plants.

Under water-deficit stress, the uptake of several nutrients declines due to reduced nutrient mass flow and diffusion (Zhao et al. 2020). Our results, in line with those of other studies, showed that the osmotic stress caused by PEG impairs the uptake of micro and macronutrients (Mouradi et al. 2016). Although AM improved nutrient levels and reduced the stress damage by expanding root depth and increasing soil access through their hyphae, the trends of the uptake, accumulation, and nutrient transfer varied in different species of plants and AM under different osmotic stress conditions. Several studies have indicated improved nutritional status of AM plants under the osmotic stress, such as N (Hashem et al. 2019), P (Zardak et al. 2018), K (Zhao et al. 2015), Cu, Zn, Fe (Abbaspour et al. 2012), and Mn (Wu and Zou 2009a, b). Since the extra radical mycelium transports water to the plant, mineral nutrients are also transported across the root-soil interface. Moreover, AM can affect the uptake of nutrients by producing different compounds; for instance, colonization of the roots by *F. mosseae* resulted in a significant increase in P uptake in the chamomile varieties of the plantlets as compared to the AM- plantlets. AM increased the amount of P absorbed by plants via increasing the activity and production of enzymes, such as phosphatase (Hu et al. 2013). One of the mineral nutrients that has been most studied in AM is P; that is because this nutrient, despite being rapidly absorbed by plants, has slow diffusion in the soil solution, which generates a depletion zone around roots (Bowles et al. 2016). When AM are formed, plant P absorption under limiting conditions is improved. It was previously shown that AM not only improved P uptake, but also increased the ‌uptake of N and nutrition in the plant through enhancing the hydraulic conductivity of the root under water-deficit stress (Gholamhoseini et al. 2013; Kong et al. 2014). Li et al. (2014) reported that under water stress, shoot P concentration decreased while root P concentration increased. In terms of P nutrition in this study, apart from root P concentration of chamomile varieties under well-watered conditions, *F. mosseae* increased P concentration in both varieties, but had a greater influence on Bod, specifically under drought conditions. The reason behind the increase in P concentration of roots under osmotic stress could be close the stomata, reduction in transpiration, imbalance of the active transport, and the disturbance in distribution; hence, P transfer from the root to shoot is reduced (Silva et al. 2009; Li et al. 2014).

Other compounds produced by AM are chelating agents, such as siderophores, which can ameliorate the uptake of micro-nutrients, like Zn and Fe in the plants (Dehghanian et al. 2018). Although certain studies have shown that the increased concentration of P by mycorrhiza has a positive effect on the Zn concentration, another reason behind the increase in the amount of Zn in the roots and shoots of mycorrhizae-inoculated plants is the rise in the diffusion-limited process of Zn (Lehmann et al. 2014). with the increase in the osmotic stress, the pores will close, which reduces transpiration and imbalances the active transport. Therefore, the transfer of nutrients reduces from the root to the shoot (Silva et al. 2009) while increasing the K concentration of the shoot by mycorrhizae rises stomatal conductance and improves the transport of nutrients from the roots to the shoots (Ruiz-Lozano et al. 1995). In the current study, root and shoot concentrations of K were higher for AM + than those for AM- seedlings at all the osmotic stress levels. It seems as though the improved plant nutrition by AM symbiosis allows cells to regulate and separate flowing ions more effectively (Giri et al. 2007). The nutrient imbalance due to osmotic stress is attributed to the effects of the stress on nutrient availability, competitive uptake, transport, or partitioning within the plants. Accumulation of K in the plant under osmotic stress is a survival policy for the plant (Wang et al. 2013). In the present work, under osmotic stress, the concentration of K increased.

To counteract osmotic stress effects, plants via an endogenous defensive mechanism consisting of different enzymatic, including POD, CAT and PPO, which is the most widely distributed osmolyte in plants (Szabados and Savouré 2010). In our experiment, antioxidant enzymes increased significantly as a function of osmotic stress, when MF colonized the chamomile varieties of the plantlets. The increased activity of POD, CAT (Uzilday et al. 2012), and PPO (Thipyapong et al. 2004) enzymes under stress condition indicated their crucial role in enduring osmotic stress. CAT is considered as the most indispensable enzyme for counteracting the hydrogen peroxide produced under stress conditions (Khanna-chopra and Selote 2007). POD is among the major H_2_O_2_-binding enzymes in cytosol and chloroplasts, whose levels also rapidly increases under water-deficit stress. Under such conditions, an increment was observed in the CAT and POD activities in diverse members of the *Asteraceae* family, such as *Silybum marianum* (Nouraei et al. 2018), *Carthamus tinctorius* L (Chavoushi et al. 2019), and *Helianthus annuus* L (Ghobadi et al. 2013). In line with our results, other studies have also reported that AM increased the levels of POD and PPO (Meddich et al. 2015; Tyagi et al. 2017) in various plants under osmotic stress. One of the reasons of the increase in POD enzyme by AM could be the expression of its encoding genes when inoculated with AM (Mustafa et al. 2017). CAT is a metalloenzyme and its activity thus depends on the availability of metal nutrients (Armada et al. 2016); meanwhile, in the present study, AM improved the uptake of metal nutrients. However, the effect of mycorrhizal inoculation on CAT enzyme levels under stress conditions was very different and depended on the plant species and even the species of mycorrhizal fungi (Wu and Zu 2009a, 2009b). The increase in CAT activity by *F. mosseae* (Amiri et al. 2015) and other species was previously reported in many plants under osmotic stress (Aalipour et al. 2020; Al-Al-Arjani et al. 2020). Our results showed a higher activity of enzymes in AM + plants during osmotic stress with respect to non-mycorrhizal plants; that is to say that AM fungi induced further effective defense mechanisms to protect the host from the detrimental effects of osmotic stress, and suggested a better resistance of colonized plants against osmotic stress.

The most common osmolytes, such as total soluble sugars and proline increased under osmotic stress, play a significant role in regulating the osmotic potential of plants to prevent the cellular damages caused by oxidative stress (Khan et al. 2015). Proline is an amino acid and can be stored in the cytoplasm, which in addition to osmotic regulation of the cell, detoxifies ROS, protects membrane integrity, and stabilizes proteins/enzymes. It also serves as one of the plant's solutions capable of reducing stress damage (Ashraf and Foolad 2007). In the current paper, an increased leaf proline level was observed by *F. mosseae* under osmotic stress.

The increase in proline content can be assigned to the effect of AM on the increase in the N concentration of the plants under water-deficit stress (Augé 2001). High N levels in the plants under osmotic stress can significantly influence the genes involved in proline biosynthesis, which finally increase proline (Monreal et al. 2007; Wang et al. 2011). In another study, an increase was reported in the total soluble sugar under drought stress conditions, which is consistent with our results (Al-Arjani et al. 2020). AM increased the level of the total soluble sugar in plants as it increased the activity of sucrose-metabolized enzymes, which had a positive and significant relationship with glucose, fructose, and sucrose contents (total soluble sugars) (Wu et al. 2013). As observed, under water-deficit stress, plant growth decreased due to reduced osmotic regulation ability, disruption of the solute uptake system, disturbance of osmotic balance, and excessive energy requirements to produce osmolytes (Munns 1993). Based on the findings of this study, a loss was observed in the dry weight of the shoots, roots, and flowers of chamomile under water-deficit stress (Baghalian et al. 2011). One of the causes of the reduced chamomile growth under stress may be the osmoregulation imbalance and the disruption in the salt absorption system or the high level of energy required for counteracting the stress (Salehi et al. 2016).

The beneficial effects of AM symbiosis on plant growth parameters and biomass production under drought stress conditions using the same system already reported for several plant species (Chitarra et al. 2016; Tsai et al. 2020). In our experiments, under osmotic stress conditions, significantly higher values were detected for AM-inoculated plants compared with those in the control plants in case of root and shoot dry weight of *F. mosseae*-colonized plants. An increment was also detected in the dry weight of the shoots, roots, and flowers of chamomile (Bączek et al. 2019) on account of the improved absorption, distribution of nutrients, the increment in proline, the total soluble sugars, and antioxidant enzymes by AM, which improved growth performance, lowered stress damage, enhanced the plant growth, and elevated dry weight (Al-Arjani et al. 2020). Chitarra et al. (2016) stated that AM mitigates drought stress by altering hormonal profiles, thereby affecting the physiology and development of the host plant and increasing dry matter.

According to the results, the dry weight of chamomile shoots, roots, and flowers reduced under drought stress (Baghalian et al. 2011). Under drought stress, plant growth declined due to the reduction in osmotic regulation, osmotic imbalance, and the requirement of excessive energy needed to cope with stress (the production of osmolytes and the disruption of the nutrient uptake) (Munns 1993). All nutrients play a vital role in plant growth; the nutrients (macro and micro) were positively correlated with the plant growth (Daur et al. 2011). The effects of each nutrient on the uptake of other nutrients are quite complex. According to the correlation shown in Fig. 6, the synergistic effect among numerous nutrients in chamomile reflects the diverse roles of these nutrients in the growth, yield, and uptake of other nutrients by chamomile; for example, sufficient Mg causes a proportional distribution of carbohydrates in the roots and shoots, promoting the chamomile root growth (He et al. 2020). In addition, Mg may affect biomass production and plant growth through proper distribution of carbohydrates and the appropriate allocation of hydrocarbons to different parts of plants (Verbruggen and Hermans 2013) or improving plants’ access to N (Haberman et al. 2019) and iron (owing to its vital role in photosynthesis) (Dong et al. 2019) with an effective role in vegetative growth and ultimately, the accumulation of plant dry weight. This is consistent with a positive and high correlation in the dry weight of chamomile with the mentioned elements. According to the results, one of the most important causes of the reduced growth of chamomile under stress conditions is the disturbed nutrients uptake (Salehi et al. 2016). The level of nutrients in the plant and the ability to uptake these nutrients are important factors in selecting the best cultivar under stress. In this regard, the Sor cultivar was almost superior to Bod in terms of both factors. On the other hand, the improved absorption and distribution of elements, as well as the increased proline, total soluble sugar, and antioxidant enzymes by mycorrhiza inoculation enhanced the growth while reducing the stress-induced damages (Al-Arjani et al. 2020). In line with previous reports (Bączek et al. 2019), mycorrhiza increased the dry weight of shoots, roots, and flowers of chamomile.

## Conclusions

Osmotic stress increased the levels of the total soluble sugar, proline, and the activity of antioxidant enzymes (CAT, POD, and PPO) in both chamomile varieties. The amount of these enzymes and osmolytes was higher in the Soroksári variety as compared with that in the Bodgol variety. Osmotic stress also reduced the uptake and transport of several nutrients from the roots to the shoots, which resulted in the decreased concentration of certain nutrients, such as N, P, Fe, and Mn, in the shoots of both varieties. Such reduction in the nutrients in the plants declined the dry weight of the plants under osmotic stress; however, the dry weight of the shoots was higher in the Soroksári variety under the control treatment and osmotic potential of − 0.4 MPa as compared with that in the Bodgol variety. AM + reduced the negative effects of osmotic stress on the plants through increasing the nutrient uptake, osmolyte contents, and activity of antioxidant enzymes.

## Data Availability

Not applicable.

## Abbreviations

AM: Arbuscular mycorrhiza
Bod: Bodgold
Sor: Soroksári
PEG: Polyethylene glycol
N: Nitrogen
P: Phosphorus
K: Potassium
Fe: Iron
Zn: Zinc
Cu: Copper
Mn: Manganese
CAT: Catalase
SOD: Superoxide dismutase
APX: Ascorbate peroxidase
ROS: Reactive oxygen species
AM +: Plants inoculated with the arbuscular mycorrhiza fungus
AM−: Not inoculated with the arbuscular mycorrhiza fungus
DW: Dry weight
O: Osmotic stress
Var: Varieties
PPO: Polyphenol oxidase
