# Correction to: Biochemical response and nutrient uptake of two arbuscular mycorrhiza-inoculated chamomile varieties under different osmotic stresses

**DOI:** 10.1186/s40529-022-00335-y

**Published:** 2022-03-05

**Authors:** Fatemeh Ebrahimi, Amin Salehi, Mohsen Movahedi Dehnavi, Amin Mirshekari, Mohammad Hamidian, Saeid Hazrati

**Affiliations:** 1 Department of Agronomy and Plant Breeding, Faculty of Agriculture, Yasouj University, Yasouj, Iran; 2 Department of Agronomy, Faculty of Agriculture, Azarbaijan Shahid Madani University, Tabriz, Iran

## Correction to: Botanical Studies (2021) 62:22 https://doi.org/10.1186/s40529-021-00328-3

Following publication of the original article (Ebrahimi et al. 2021), errors were identified in Tables 1 and 4, The data are reversed in Tables 1 and 4 and the right decimal place is moved to the left decimal place.

**Table 1:** Analysis of variance of *osmotic stress*, arbuscular mycorrhiza and variety and their interactions effects on nutrients uptake in chamomile

Source of variation	df	Shoot concentration							Root concentration						
		N	P	K	Zn	Fe	Cu	Mn	N	P	K	Zn	Fe	Cu	Mn
O	2	2114.47**	29.81**	1565.17**	65040.23**	13444.14**	2636.89**	3939.09**	164.26*	131.61**	29.66**	51988.02**	428.06**	4332.16**	2803.51**
AM	1	181.96**	25.66**	208.29**	4504.68**	1906.38**	2813.66**	3236.61**	41.25**	26.8**	506.32*	3663.02^ns^	453.25**	1328.25**	1652.08**
Var	1	201.80*	5.32**	55.60**	500.52**	550.13**	338.66**	413.24*	1.28**	26.1**	606.16**	1518.65**	56.42**	109.50**	42.18**
O × AM	2	13.38**	0.65**	0.04^ns^	289.45**	43.88**	9.66^ns^	26.66^ns^	3.32**	0.66^ns^	31.50**	25.52^ns^	32.16**	4.03^ns^	115.65**
O × Var	2	54.56**	2.69**	22.20**	111.87*	129.06**	60.60^ns^	196.69*	0.21^ns^	3.69**	18.66**	465.00**	19.14**	22.68*	22.26**
AM × Var	1	1.58**	0.96**	2.98^ns^	133.33^ns^	1.16^ns^	141.69*	269.56*	0.62*	0.30^ns^	19.65**	4.68^ns^	0.13^ns^	1.16^ns^	33.33**
O × AM × Var	2	5.46**	1.06**	5.80^ns^	28.28^ns^	68.35**	50.39^ns^	80.29^ns^	0.16^ns^	0.35^ns^	0.69^ns^	4.68^ns^	0.91^ns^	5.06^ns^	6.38^ns^
Error	36	0.223	0.019	2.46	34.11	1.16	28.68	45.64	0.146	0.30	2.62	14.40	1.51	4.46	4.06
CV%		2.05	1.20	3.15	3.69	1.34	8.34	10.23	4.63	5.08	4.64	3.12	3.26	3.98	3.66

O: *Osmotic stress*, Var: Varieties, AM: Arbuscular mycorrhizal inoculation, ns: non-significance at *P* ≤ 0.05; *P ≤ 0.05; **P ≤ 0.01, statistical significance

**Table 4:** Analysis of variance of *osmotic stress*, arbuscular mycorrhiza and variety and their interactions effects on osmolytes, activity of antioxidant enzymes (catalase (*CAT*), peroxidase (POD), polyphenol oxidase (PPO)), and shoot and root dry weights

Source of variation	df	Proline	Total soluble sugar	CAT	POD	PPO	Shoot dry weight	Root dry weight
O	2	136.50**	36248.39**	3864.92**	2.62**	217.80**	1.09**	0.002**
AM	1	16.54**	2693.25**	281.51**	0.13**	36.63**	0.083**	0.008**
Var	1	30.56**	753.58**	210.45**	0.34**	33.87**	0.015**	0.00086**
O × AM	2	1.12**	419.88**	26.07**	0.009**	4.40**	0.017**	0.00083**
O × Var	2	0.04**	79.14**	43.14**	0.09**	6.19**	0.148**	0.0001**
AM × Var	1	0.44**	6.87^ns^	2.59^ns^	0.01**	1.35**	0.0068**	0.0003**
O × AM × Var	2	0.43**	17.17^ns^	8.18**	0.013**	0.9**	0.0058**	0.00009^ns^
Error	36	0.006	7.40	0.832	0.0003	0.126	0.0001	0.00003
CV%		1.32	2.92	4.41	3.46	5.08	2.25	5.88

O: *Osmotic stress*, Var: Varieties, AM: Arbuscular mycorrhizal inoculation, ns: non-significance at *P* ≤ 0.05; *P ≤ 0.05; **P ≤ 0.01, statistical significance

The correct tables are given below.
